# Application and efficacy of Retzius-sparing robotic-assisted radical prostatectomy

**DOI:** 10.3389/fsurg.2026.1774133

**Published:** 2026-02-17

**Authors:** Zhicheng Zeng, Faxiong Zhang, Neng Luo, Yalin Li, Xiaofeng Zou, Hui Xu

**Affiliations:** Department of Urology, First Affiliated Hospital of Gannan Medical University, Ganzhou, China

**Keywords:** prostate tumor, prostatectomy, Retzius space preservation, robot-assisted laparoscopy, urinary continence

## Abstract

**Background and objective:**

The results of comparing standard Robotic-assisted Radical Prostatectomy (S-RARP) and Retzius-sparing Robotic-assisted Radical Prostatectomy (RS-RARP) in treating early urinary continence (UC), oncological outcomes, operation time, and perioperative morbidity in patients with clinically localized prostate cancer were analyzed.

**Methods:**

A retrospective analysis was conducted on the clinical data of 120 cases of RS-RARP performed at the First Affiliated Hospital of Gannan Medical University from October 2019 to March 2025. Among them, 60 cases underwent standard Robotic-assisted Radical Prostatectomy (S-RARP), and 60 cases underwent Retzius-sparing Robotic-assisted Radical Prostatectomy (RS-RARP). The clinical data, perioperative indicators, and surgical outcomes of the two groups of patients were compared.

**Results:**

All 120 surgeries were successfully completed. The operation time (mean ± SD) of the S-RARP group was (138.97 ± 47.24) minutes, and that of the RS-RARP group was (150.78 ± 51.72) minutes. There was no statistically significant difference (*P* > 0.05). The intraoperative blood loss (IQR) of the S-RARP group was 100.00 (237.50) mL, and the incidence of perioperative complications was 8.33% (5/60); for the RS-RARP group, it was 100.00 (150.00) mL and 6.67% (4/60), and the differences between the two groups were not statistically significant (all *P* > 0.05). The positive rate of surgical margins in the S-RARP group was 30% (18/60), and that in the RS-RARP group was 35% (21/60), and the difference was not statistically significant (*P* > 0.05). Comparisons of the immediate, first-week, and first-month urinary continence recovery rates between the two groups showed that the RS-RARP group was superior to the S-RARP group, and the differences were statistically significant (all *P* < 0.01); however, the third-month urinary continence recovery rate showed no statistically significant difference (*P* = 0.057).

**Conclusions:**

Retzius-sparing Robotic-assisted Radical Prostatectomy is beneficial for improving the early recovery of urinary incontinence in patients.

## Introduction

The earliest recorded prostate cancer surgery dates back over 100 years. In 1901, the Frenchman Proust performed a transperineal prostatectomy, and in 1904, the American Young performed a prostate cancer surgery (1). Due to the limitations of medical technology at that time, the mortality risk of radical prostatectomy for radical prostate cancer surgery was as high as 30% (2). Starting from the 1940s, radical prostatectomy through the posterior pubic approach began to be applied in clinical practice. In the 1980s, Professor Walsh conducted research on the pelvic floor anatomy of the prostate and proposed a posterior pubic nerve-preserving prostatectomy, elevating the prostate cancer surgery technology to a new level. Before this, the S-RARP surgery had become the classic method for radical prostatectomy (3).

Although the radical prostatectomy through the posterior pubic approach significantly reduced intraoperative bleeding and improved postoperative urinary control and sexual function, 7% of patients still needed diapers one year after the operation (3). To achieve more minimally invasive and better functional recovery, urologists have been exploring more treatment methods and new technologies. After 1990, laparoscopic technology was applied in the field of urology. In 1997, Schuessler et al. (4) reported laparoscopic radical prostatectomy (LRP), and by 2000, German Binder and Kramer (5) first performed robot-assisted laparoscopic radical prostatectomy, using the clear 3D vision and flexible robotic arms of the robot to achieve better surgical results and develop the VIP technology. However, whether it is the classic open nerve-preserving radical prostatectomy or the surgical techniques of the robot era, both patients and doctors still face the problem of early urinary incontinence after the operation. The proportion of postoperative urinary incontinence one month after the operation varies from 10% to 50%, which is one of the main problems affecting the quality of life of patients after the operation (6). In 2010, Professor Bocciardi from Italy proposed a new robotic surgical approach, opening the peritoneum behind the bladder and in front of the rectum in the Douglas fossa, and completely removing the prostate. This technique was named the robot-assisted laparoscopic radical prostatectomy with Retzius space preservation (Retzius-sparing RARP, RS-RARP), and is generally referred to as the posterior approach prostatectomy. The posterior approach has better immediate urinary control and 3-month and 6-month urinary control than traditional robot-assisted prostatectomy (7). By 2020, the EAU Prostate Cancer Diagnosis and Treatment Guidelines recommended the posterior approach surgery as one of the surgical methods for prostate cancer (8).

The posterior approach surgery has obvious advantages in immediate and early urinary control after the operation. However, when transitioning from the standard anterior approach surgery to the posterior approach surgery, surgeons often face difficulties such as limited surgical space, few surgical anatomical landmarks, and changes in surgical habits. Therefore, at present, the adoption rate of posterior approach prostate cancer surgery in Europe is not high, and the number of units and doctors performing posterior approach surgery in China is relatively small, and it is even less frequently carried out routinely. The center where I work began to perform posterior approach prostate cancer surgery in 2023, and so far, we have completed over 100 posterior approach robot-assisted laparoscopic radical prostatectomies, and now we have routinely carried out posterior approach surgery. Therefore, in this study, we compared two groups of prostate cancer patients who received different surgical approaches to evaluate their surgical outcomes.

## Materials and methods

After obtaining approval from the ethics committee, a retrospective analysis was conducted on patients who underwent radical prostatectomy at the First Affiliated Hospital of Gannan Medical University from October 2019 to March 2025. Inclusion criteria: (1) No severe cardiovascular, pulmonary or systemic diseases, and physically capable of undergoing surgery; (2) Expected survival time >10 years and willing to undergo postoperative follow-up; (3) Diagnosed with prostate cancer through pathological examination; (4) Local early-stage or partially locally advanced patients with no tumor metastasis; (5) No radiotherapy or chemotherapy before the surgery. Exclusion criteria: (1) Having contraindications for laparoscopic surgery (such as tumor metastasis, hematological diseases, cardiac or pulmonary dysfunction, etc.); (2) Complicated with mental disorders, cognitive impairments, etc., and unable to cooperate with treatment; (3) Having urinary incontinence, infections, etc. before the surgery; (4) Complicated with other primary tumors or other severe underlying diseases; (5) Having a history of complex pelvic surgeries in the past. A total of 120 patients operated by one surgical team were divided into two groups. Patients with prostate cancer before 2023 were classified into Group 1 based on inclusion and exclusion criteria. Patients with prostate cancer after 2023 were divided into Group 1 and Group 2 based on the different surgical approaches. The 60 patients in the first group underwent surgery using the S-RARP technique, and the 60 patients in the second group underwent surgery using the RS-RARP technique. The collected clinical data included height, weight, age, BMI, preoperative neoadjuvant therapy, prostate-specific antigen (PSA) level, pathological prostate volume, clinical stage; the collected perioperative and postoperative follow-up data included total hospital stay, operation time, hemoglobin difference, postoperative drainage situation, positive rate of surgical margins, perioperative complications, postoperative urinary incontinence (considered as urinary control recovery if using no more than 1 pad within 24 h) (8), biochemical recurrence (BCR) situation (continuous two postoperative PSA levels >0.2 ng/mL). On the day of extubation, patients were reminded by phone to come to the outpatient clinic to observe urinary incontinence, and the urinary control status and postoperative PSA levels of the patients were recorded at 1 week, 1 month, 3 months and 6 months after the operation, with a median follow-up time of 11 (6–23) months.

### Surgical technique

In the practice of posterior approach surgery, our team, through the practice and reflection of posterior approach surgery, proposed a surgical strategy of dividing the prostate into three layers, and achieved good surgical results and excellent urinary control efficacy. We have realized that by dividing the prostate into three layers, it is possible to better locate the surgical plane, establish surgical anatomical landmarks, and avoid surgical complications. The method is as follows:

- Anesthesia and position: The patient is given general anesthesia via endotracheal intubation combined with intravenous administration. Arterial cannulation is used to monitor dynamic invasive arterial pressure and dynamic monitoring of CO2 partial pressure. Deep venous cannulation maintains a clear venous channel. The patient is placed in the Trendelenburg position, with the head lower than the feet (approximately 25°–30°), and is properly fixed on the operating table. The two lower limbs are separated and placed flat, elastic stockings are worn to prevent deep vein thrombosis of the lower limbs, the shoulder is supported with a shoulder brace to prevent brachial plexus nerve injury, the head is slightly raised, and eye closure film is applied for disinfection and surgical draping before inserting the urinary catheter.
- Position of the cannula (Davanci XI model Trocar): The lens cannula puncture hole is located 1 cm below the umbilicus, and the mechanical arms R1, R2, and R3 are all located 1–2 cm above the umbilicus (approximately 20 cm from the pubic symphysis) and distributed in a straight line. The 1st arm of the right-handed surgeon's cannula puncture hole (R1) is located 12 cm to the left of the midline, the 2nd (R2) mechanical arm cannula puncture hole is located 10 cm to the right of the midline, and the 3rd mechanical arm cannula puncture hole (R3) is located outside the 2nd mechanical arm cannula puncture hole (R2), approximately 8 cm away from the 2nd mechanical arm cannula puncture hole (R2). The 12 mm assistant cannula puncture hole is located on the inner side of the 1st mechanical arm cannula puncture hole (R1), between the midline and the R1 on the head side. If necessary, an additional 5 mm assistant cannula hole can be added between the lens cannula puncture hole and the 2nd (R2) mechanical arm cannula puncture hole. The 1st mechanical arm instrument is a robot Maryland bipolar forceps or Cardier bipolar forceps, the 2nd mechanical arm instrument is a robot Monopolar single-pole cutter or needle holder, and the 3rd mechanical arm instrument is a robot Prograsp forceps. Due to the relatively small space of posterior approach surgery, the main operating instrument of the 2nd mechanical arm (Maryland bipolar forceps) can be swapped with the Prograsp forceps of the 3rd mechanical arm, which is responsible for traction, to obtain greater instrument mobility.
- Robot positioning and layout of the operating room: After the puncture and cannula placement are completed, adjust the position, use the extension line of the puncture point of the lens arm and the target area as the reference line, and be commanded by one assistant, the circulating nurse pushes the robot cart (Patient Cart) into position for docking. Ensure that the puncture point of the lens arm, the lens arm, and the central column are in a straight line. Connect each mechanical arm with each puncture point's cannula (Trocar), insert the lens at a 30° upward angle and fix it into the abdominal cavity, connect the Monopolar single-pole cutter and Maryland bipolar forceps with the 1st and 2nd arms respectively, and move the instruments into the surgical area under direct vision and fix them. Connect the Prograsp forceps with the 3rd arm, move it into the left side of the abdominal cavity outside the surgical area and fix it for future use. Thus, the robot cart is docked.

Robot-assisted laparoscopic posterior approach radical prostatectomy:

- Open the peritoneum. Separate the adhesion between the sigmoid colon and the pelvic cavity, expose the Douglas pouch, make a horizontal incision about 5 cm long 2 cm above the rectum and the abdominal fold, search for and expose the vas deferens and seminal vesicle, and free along its surface to the bottom of the prostate.
- Separate the posterior layer between the prostate and the Denounvillier fascia. Using the fourth arm to lift the vas deferens upwards, change the observation mirror to 300° upwards, expose the junction where the Denounvillier fascia fuses with the prostate, and perform a blunt dissection to remove the Denounvillier fascia downward, entering the posterior layer between the prostate and the Denounvillier fascia (fascial technique) or entering the anterior layer of the rectal anterior fat (fascial external technique), proceeding to separate along this layer to the lateral plane and the posterior part of the prostate apex.
- Free the vas deferens and seminal vesicle. Dissect and sever both sides of the vas deferens, completely free the seminal vesicle, and perform bipolar electrocoagulation on the supplying vessels.
- Separate and enter the lateral plane of the prostate. Pull the seminal vesicle to the opposite side, using sharp + blunt methods with an electric cutter, gradually advancing to the surface of the avascular area of the prostate capsule, push the nerve and blood vessel bundles outward, enter the fascial internal or inter-fascial plane, or freely separate to the pelvic fascial level on the outside of the prostate, open the pelvic fascia, and expose the nerve and blood vessel bundles of the prostate (fascial external), proceed to advance along the lateral plane of the prostate to the prostate apex and further dissection towards the anterior side of the prostate.
- Dissect to the bladder neck, sever the bladder neck, free the lateral plane of the prostate, then perform blunt separation towards the anterior side of the bladder smooth muscle, visible the shape of the prostate bladder neck connection, pull the airbag to confirm and then sever the bladder neck.
- Separate and enter the anterior plane between the prostate and the anterior fibrous matrix band, dislocate along the prostate capsule to the prostate apex.
- Sever the urethra, rotate the prostate, dislocate along the posterior, lateral prostate capsule to the apex, carefully identify the prostate apex, use scissors to sever the urethra, remove the specimen.
- Suture the bladder neck and urethra. Use 3–0, 5/8 arc double needle barbed thread, start from 12 o'clock at the bladder neck, respectively ligate the urethra on both sides, make a knot at 6 o'clock, after the ligation is completed, change the urinary catheter. Inject 100 mL of water, confirm no urine leakage.
- Package the specimen, check the surgical field and hemostasis, remove the specimen, leave a drainage tube. Suture all cannula punctures and incisions, and bandage with sterile dressing.

Robot-assisted laparoscopic anterior approach radical prostatectomy:

- Cut the pelvic fascia of both sides of the prostate, expose the prostate on both sides, clear the fat below the pubic symphysis, separate and sever the pubic-prostate ligament.
- Fully free the deep dorsal vein complex of the penis, 2-0 micro bridge thread ″8″ stitch, pull the urinary catheter, confirm the ultrasound knife incision of the prostate bladder connection, expose the internal opening of the bladder, continue to cut and dislocate the bilateral seminal vesicles, both sides of the vas deferens.
- Sever the vas deferens, lift both sides of the vas deferens and seminal vesicles, dislocate along the lower gap to the prostate apex, cut the anterior wall of the urethra with scissors, withdraw the urinary catheter, continue to sever the posterior wall of the urethra, perform forward and backward dissection to completely remove the prostate.
- Use 2-0 suture, start from 9 o'clock at the bladder neck, continuously ligate the bladder neck and urethra, after the ligation is completed, change the urinary catheter. Inject 100 mL of water, confirm no urine leakage.
- Package the specimen, check the surgical field and hemostasis, remove the specimen, leave a drainage tube. Suture all cannula punctures and incisions, and bandage with sterile dressing.

### Statistical analysis

Statistical analysis was conducted using SPSS 27.0 software to test whether the research data followed a normal distribution. Continuous variables with a normal distribution were presented as mean ± standard deviation, and comparisons between groups were performed using *t*-tests; continuous variables with non-normal distribution were expressed as median (interquartile range), and comparisons between groups were conducted using the Mann–Whitney U test; categorical variables were presented as percentages (%), and comparisons between groups were performed using the chi-square test. *P <* 0.05 was considered statistically significant. Using univariate and multivariate logistic regression analysis, the relationship between independent predictors and urinary incontinence in patients was explored, and a sensitivity analysis was conducted by restricting the time window.

## Results

### Baseline information

A total of 120 patients underwent the surgery, among which 60 cases received the posterior approach and 60 cases received the anterior approach. All procedures were successfully completed without any cases of conversion to open surgery, no cases of intraoperative or postoperative blood transfusion, and no serious intraoperative or postoperative complications occurred. The summary of their clinical characteristics is shown in Table 1. The age (mean ± SD) of the two groups was (69.83 ± 7.14) and (70.33 ± 7.46) years old, respectively, the BMI (mean ± SD) was (23.74 ± 2.49) and (23.79 ± 2.88) kg/m^2^, respectively, the PSA (IQR) was 8.54 (21.37) and 7.87 (18.07) ng/mL, respectively, and the prostate volume (mean ± SD) was (44.36 ± 17.93) and (42.50 ± 19.69) mL, respectively. The *n* (%) of patients with clinical T stage <T3 in the two groups were 42 cases (70.00%) and 41 cases (68.33%), respectively. The *p* values of age, PSA, BMI, prostate volume, and clinical T stage in the two groups were all >0.05, and the differences were not statistically significant.

**Table 1:** Clinical data.

Characteristics	RS-RARP(*n* = 60)	S-RARP(*n* = 60)	*P*
Age(mean ± SD)	69.83 ± 7.14	70.33 ± 7.46	0.708
BMI(mean ± SD)	23.74 ± 2.49	23.79 ± 2.88	0.930
PSA(IQR)	8.54 (21.37)	7.87 (18.07)	0.923
Prostate volume(mean ± SD)	44.36 ± 17.93	42.50 ± 19.69	0.593
Clinical T stage, n (%)			
<T3	42 (70.00)	41 (68.33)	0.843
≥T3	18 (30.00)	19 (31.67)	

BMI, body mass index; IQR interquartile range; PSA, prostate-specifc antigen; S-RARP, standard robot-assisted radical prostatectomy; RS-RARP, Retzius-sparing robotic-assisted radical prostatectomy; IQR, interquartile range; SD, standard deviation.

### Perioperative outcome

As shown in Table 2, the operation time (mean ± SD) of the two groups of patients was (150.78 ± 51.72) and (138.97 ± 47.24) minutes respectively, the change in hemoglobin (mean ± SD) was (12.97 ± 7.46) and (13.5 ± 7.30) g/L respectively, the number of perioperative complications [n (%)] was 4 cases (6.67) and 5 cases (8.33) respectively, among which 3 patients developed postoperative pulmonary infection, 2 patients developed respiratory failure after surgery, 2 patients developed sepsis after surgery, and 2 patients developed intestinal obstruction after surgery. After symptomatic treatment, all patients improved. The postoperative hospital stay (mean ± SD) of the two groups of patients was (7.50 ± 2.60) and (7.10 ± 2.27) days respectively. The intraoperative blood loss (IQR) was 100.00 (150.00) and 100.00 (237.50) mL respectively, the postoperative pathological stage *n* (%) was T1c 7 cases (11.67) and 2 cases (3.33), T2a 12 cases (20.00) and 14 cases (23.33), T2b 6 cases (10.00) and 9 cases (15.00), T2c 19 cases (31.67) and 20 cases (33.33), T3a 2 cases (3.33) and 4 cases (6.67), T3b 14 cases (23.33) and 11 cases (18.33). The positive rate of surgical margins *n* (%) was 21 cases (35) and 18 cases (30) respectively. The *p* values of the operation time, change in hemoglobin, perioperative complications, intraoperative blood loss, postoperative pathological stage, and positive rate of surgical margins of the two groups of patients were all >0.05, and the differences were not statistically significant.

**Table 2:** Perioperative indicators.

Characteristics	RS-RARP (*n* = 60)	S-RARP (*n* = 60)	*P*
Operation time(mean ± SD)	150.78 ± 51.72	138.97 ± 47.24	0.194
hemoglobin change(mean ± SD)	12.97 ± 7.46	13.5 ± 7.30	0.693
Perioperative complications,n (%)	4 (6.67)	5 (8.33)	5 (8.33)
Postoperative hospital stay(mean ± SD)	7.50 ± 2.60	7.10 ± 2.27	1.0000.608
Intraoperative bleeding(IQR)	100.00 (150.00)	100.00 (237.50)	0.277
Pathological stage, *n* (%)			0.488
T1c	7 (11.67)	2 (3.33)	
T2a	12 (20.00)	14 (23.33)	
T2b	6 (10.00)	9 (15.00)	
T2c	19 (31.67)	20 (33.33)	
T3a	2 (3.33)	4 (6.67)	
T3b	14 (23.33)	11 (18.33)	
PSM	21 (35)	18 (30)	0.559

PSM, positive surgical margin.

### Urinary continence outcome

The data on postoperative urinary control of the patients are shown in Table 3. The group with the posterior approach showed statistically significant differences in early urinary control. There were significant differences in immediate urinary control (71.7% vs. 16.7%, *p* < 0.01), first-week urinary control (80.0% vs. 33.3%, *p* < 0.01), and first-month urinary control (90.0% vs. 46.7%, *p* < 0.01). However, no significant difference was found at third months (100.0% vs. 91.7%, *p* = 0.057).

**Table 3:** Operation result.

Urinary continence *n* (%)	RS-RARP (*n* = 60)	S-RARP (*n* = 60)	*P*
Immediate continence	43 (71.7)	10 (16.7)	<0.01
At first week	48 (80)	20 (33.3)	<0.01
At first month	54 (90)	28 (46.7)	<0.01
At third month	60 (100)	55 (91.7)	0.057

### Univariate logistic regression and multivariate logistic regression analysis

The data of univariate and multivariate logistic regression analyses for immediate urinary continence in patients are presented in Table 4. To help determine whether the urinary continence advantage observed by RS-RARP is independent of potential confounding factors such as age, prostate volume, pathological stage, and learning curve effect, we conducted a logistic regression analysis. When dealing with the learning curve effect, we regarded it as a continuous variable and recorded it as the cumulative number of surgeries completed by the surgical team. The univariate logistic regression analysis indicated that RS-RARP (OR = 0.08; 95% CI: 0.03–0.19; *p* < 0.01), clinical stage (≥T3) (OR = 2.94; 95% CI: 1.19–7.27; *p* = 0.02), and prostate volume (OR = 1.04; 95% CI: 1.01–1.08; *p* = 0.03) were risk predictors for immediate urinary continence. After eliminating multicollinearity, the surgical approach, age, prostate volume, pathological stage, and learning curve effect were included in the multivariate logistic regression analysis. The data showed that RS-RARP (OR = 0.06; 95% CI: 0.02–0.16; *p* < 0.01), clinical stage (≥T3) (OR = 5.23; 95% CI: 1.66–16.47; *p* < 0.01), and prostate volume (OR = 1.05; 95% CI: 1.01–1.10; *p* = 0.04) were independent predictors of immediate urinary continence.

**Table 4:** Univariate logistic regression and multivariate logistic regression analysis of immediate continence.

Variable	Univariate analysis		Multivariate analysis	
	OR (95% Cl)	*p*-Value	OR (95% Cl)	*p*-Value
RS-RARP	0.08 (0.03–0.19)	<0.01	0.06 (0.02–0.16)	<0.01
S-RARP				
Age	1.01 (0.96–1.06)	0.72	1.00 (0.94–1.06)	0.95
Prostate volume	1.04 (1.01–1.08)	0.03	1.05 (1.01–1.10)	0.04
Clinical T stage				
<T3				
≥T3	2.94 (1.19–7.27)	0.02	5.23 (1.66–16.47)	<0.01
Learning curve	1.00 (0.98–1.02)	0.97	1.00 (0.97–1.03)	0.92

### Sensitivity analysis

Since the S-RARP cases can be traced back to 2019, while the RS-RARP cases began to be routinely implemented in 2023, this has led to the possibility that the primary outcome indicators may be affected by overall medical technological advancements, surgical experience, etc. Therefore, our analysis is limited to the overlapping period from 2023 to 2025. In this subgroup, a total of 30 patients with S-RARP and 60 patients with RS-RARP were included. Baseline data comparison, outcome indicator comparison, and multivariate logistic regression analysis were re-conducted (Table 5). After restricting the time window (2023–2025), the baseline characteristics of the two groups remained balanced (all *P* > 0.05). Consistent results were obtained in the multivariate logistic regression analysis and the primary outcome indicators (in the Supplementary Tables S1, S2).

**Table 5:** Sensitivity analysis.

RS-RARPvs S-RARP	Queue period	sample size	Immediate continence	*P*
Mainly analyze	2019–2025	60 vs. 60	43 (71.7) vs. 10 (16.7)	<0.01
Sensitivity analysis	2023–2025	60 vs. 30	43 (71.7) vs. 6 (20)	<0.01

## Discussion

With the improvement of living standards, people naturally develop a desire for a higher quality of life. More and more prostate cancer patients are no longer satisfied with tumor control alone, but also demand to retain sexual function and early recovery of urinary control after surgery. For urologists, our thinking needs to shift to meeting patients' needs for urinary control and sexual function recovery without affecting tumor control. In this study, we retrospectively analyzed the case data of prostate cancer patients who underwent RS-RARP and RARP, and found that the method of preserving the Retzius space was related to the early recovery of urinary control.

In 2010, Professor Bocciardi from Italy proposed a new robotic surgical approach. The peritoneum was opened in the Douglas fossa behind the bladder and in front of the rectum, and the prostate was completely removed. This surgical approach is different from the traditional anterior approach surgery. The traditional anterior approach surgery uses the bladder as a landmark, separates the anterior wall of the bladder and enters the posterior space of the pubic bone (Vattikuti-style as an example), then removes the prostate. This surgical method will damage some important structures in the anterior cavity, including nerve and blood vessel bundles, Aphrodite veil, pelvic fascia, Santorini plexus, pubocervical ligament, and all the structures that are believed to play a role in maintaining erection and sexual function (9–11). The posterior approach Douglas space follows a complete intra-fascial plane and does not dissect the anterior cavity (12). It bypasses the related anatomical structures related to urinary incontinence, such as the Retzius space. Therefore, this technique was named the robot-assisted laparoscopic radical prostatectomy with Retzius preservation (Retzius-sparing RARP, RS-RARP), and is generally referred to as posterior approach prostatectomy. This technique simulates the earliest transperineal approach prostatectomy. Prostatectomy is performed through the intra-fascial or inter-fascial plane. Due to its complete preservation of important structures in the anterior cavity and more preservation of the bladder neck muscles, good urinary control was achieved after surgery. By 2020, the EAU prostate cancer diagnosis and treatment guidelines recommended posterior approach surgery as one of the surgical methods for prostate cancer.

The superior performance of RS-RARP in urinary control has led more and more urologists to try this surgical method. The related research has also increased. In terms of urinary control, multiple centers' high-quality randomized controlled study data have confirmed that RS-RARP has significant advantages in immediate and early urinary control. The results of Qiu X et al.'s one-year follow-up in a randomized controlled, single-blind trial showed that one week after extubation, the urinary control of the posterior approach group was significantly higher than that of the anterior approach group (*p* < 0.05) (13). The study by Mani et al. showed that the early recovery of the posterior approach (Bocciardi) approach had significant advantages, but there was no significant difference between the two groups after more than 6 months (14). Asimakopoulos A D et al.'s study also showed that the posterior approach was superior to the traditional robotic prostatectomy in immediate urinary control, at 3 months and 6 months after surgery (15). Among the 60 patients in the posterior approach group of this study, the immediate urinary continence rate was 71.7%, significantly higher than that of the anterior approach group. Moreover, the urinary continence rates within 1 week and 1 month were also higher than those of the anterior approach group. Similar conclusions were drawn, which were consistent with those of other high-quality studies (16–18).

The posterior approach surgery has obvious advantages in immediate and early urinary control after surgery. However, when transitioning from the standard anterior approach surgery to the posterior approach surgery, surgeons often face difficulties such as limited surgical space, few surgical anatomical landmarks, and changes in surgical habits. Therefore, at present, the adoption rate of posterior approach prostate cancer surgery in Europe is not high (19–21). In China, the number of units and doctors performing posterior approach surgery is relatively small, and it is even less frequently carried out. The center where our team is located began to implement posterior approach prostate cancer surgery in 2023. Through technological improvement, up to now, we have successfully completed over 100 posterior approach robot-assisted laparoscopic radical prostatectomy surgeries. We have now routinely carried out posterior approach surgeries and have become one of the early and few units in China to routinely perform such surgeries. In the practice of posterior approach surgeries, our team, through practical experience and reflection on posterior approach surgeries, proposed a surgical approach for the prostate divided into three layers, and achieved good surgical results and excellent urinary control effects. We have realized that by dividing the prostate into three layers, it is possible to better find the surgical planes, establish surgical anatomical landmarks, and avoid surgical complications. Now, we introduce the method as follows: We divide the globular prostate into three layers. The first layer is the posterior plane between the Denonvillier fascia and the rectum. The second layer is the lateral plane between the prostate capsule and the fascia. The third layer is the anterior plane between the prostate and the anterior fibromuscular stroma band. The vas deferens and seminal vesicle are the first important anatomical landmark during the surgery. After exposing the Douglas fossa and revealing the vas deferens and seminal vesicle, we do not first free the seminal vesicle and vas deferens, but instead, along their surface, we free them into the bottom of the prostate. The anterior plane of the Denonvillier fascia and the anterior plane of the rectum is a natural loose anatomical plane. Using the robotic arm to pull the vas deferens and performing blunt dissection of the Denonvillier fascia to enter the posterior plane between the prostate and the Denonvillier fascia (technique within the fascia) or into the anterior plane of the rectum (technique outside the fascia), then we free the vas deferens and seminal vesicle gland. The surgical difficulty lies in transitioning from the first layer to the second layer. This is also the part where NVB attaches and blood vessels enter the prostate. Using the anatomical “scalpel” technique to handle the area where NVB enters the prostate, to achieve precise coagulation, it is recommended to set the energy level at less than 30 watts of electrocoagulation, with a short duration (less than 1 s), and try to avoid using electrocoagulation near the nerve and blood vessel bundles (5−10 mm). The bladder neck is another important anatomical landmark during the surgery. The urinary catheter helps to better locate the bladder neck. By blunt and sharp dissection of the bladder prostate muscle and the muscles around the bladder neck, the shape of the bladder neck can be well revealed, regardless of the size of the prostate. The posterior approach can retain all the internal urethral sphincters of the prostate, while the anterior approach may sever the muscle of the bladder-prostate connection at 2–6 o'clock and 8–12 o'clock. The male urinary control mechanism mainly relies on the muscle tension of the urethral internal sphincter and the pelvic floor muscle complex and the function of the pubocervical urethral ligament suspension. Therefore, the posterior approach has better early urinary control function. By dissecting the seminal vesicle and vas deferens, revealing the Denonvillier fascia, through the method of transitioning from the first layer to the second layer, the lateral plane upward to the bladder neck, the anterior plane between the Denonvillier fascia and the rectum, and the lateral plane of the prostate, the anterior plane between the prostate and the anterior fibromuscular stroma band can be well completed the surgery. This study has many limitations. Firstly, it is a non-randomized, single-center, retrospective study, which inevitably has selection bias. Secondly, the sample size involved is small and the follow-up time for tumors is short. The results of this study still need to be verified by larger sample sizes and further randomized controlled studies.

## Conclusion

In conclusion, robot-assisted laparoscopic posterior approach radical prostatectomy has a significant advantage over the traditional anterior approach in terms of early urinary control recovery, but a longer follow-up period is needed to confirm its oncological safety.

## Data Availability

The original contributions presented in the study are included in the article/Supplementary Material, further inquiries can be directed to the corresponding author/s.
