# Supplementary material for: Application and efficacy of Retzius-sparing robotic-assisted radical prostatectomy
Source: Front Surg. 2026 Feb 17;13:1774133. doi: 10.3389/fsurg.2026.1774133 (PMC12953507; doi:10.3389/fsurg.2026.1774133)
Supplement: Supplementary file 1 [file Table1.docx]

supplementary table 1

|  | RS-RARP(n=60) | S-RARP(n=30) | *P* |
| --- | --- | --- | --- |
| Age(mean±SD) | 69.83±7.14 | 70.07±7.75 | 0.887 |
| BMI(mean±SD) | 23.74±2.49 | 23.73±3.02 | 0.978 |
| PSA(IQR)  Prostate volume(mean±SD) | 8.54（21.37）  44.36±17.93 | 8.92(28.58)  46.40±7.64 | 0.669  0.329 |
| Clinical T stage,n（%）  < T3 | 42（70.00） | 20(66.67) | 0.747 |
| ≥T3  Operation time(mean±SD)  hemoglobin change(mean±SD)  Perioperative complications,n（%）  Postoperative hospital stay(mean±SD)  Intraoperative bleeding(IQR)  Pathological stage,n（%）  T1c  T2a  T2b  T2c  T3a  T3b  PSM  Urinary continence  Immediate continence  At first week  At first month  At third month | 18（30.00）  150.78±51.72  12.97±7.46  4（6.67）  7.50±2.60  100.00（150.00）  7（11.67）  12（20.00）  6（10.00）  19（31.67）  2（3.33）  14（23.33）  21（35）  43（71.7）  48（80）  54（90）  60（100） | 10(33.33)  142.7±46.85  13.67±6.88  3(10.00)  7.47±2.64  125(250.00)  2(6.67)  5(16.67)  5(16.67)  8(26.67)  3(10.00)  7(23.33)  10(33.33)  6(20)  11(36.7)  15(50)  28(93.3) | 0.473  0.693  0.682  0.800  0.196  0.713  1.00  ＜0.01  ＜0.01  ＜0.01  0.109 |

supplementary table 2

| Variable | Univariate analysis | | Multivariate analysis | |
| --- | --- | --- | --- | --- |
|  | OR (95% Cl) | *p*-Value | OR (95% Cl) | *p*-Value |
| RS-RARP  S-RARP | 0.10(0.03–0.28) | <0.01 | 0.07(0.02–0.25) | <0.01 |
| Age | 1.02(0.96–1.08) | 0.57 | 1.01(0.94–1.09) | 0.75 |
| Prostate volume | 1.05(1.01–1.10) | 0.03 | 1.06(1.01–1.13) | 0.04 |
| Clinical T stage  < T3  ≥T3  learning curve | 5.18(1.89–14.21)  1.02(0.99–1.04) | <0.01  0.24 | 7.47(2.20–25.37)  0.98(0.95–1.02) | <0.01  0.40 |
